# Thyroidectomy for Painful Thyroiditis Resistant to Steroid Treatment: Three New Cases with Review of the Literature

**DOI:** 10.1155/2015/138327

**Published:** 2015-06-02

**Authors:** Enrico Mazza, Francesco Quaglino, Adolfo Suriani, Nicola Palestini, Cristina Gottero, Renzo Leli, Stefano Taraglio

**Affiliations:** ^1^Endocrinology and Metabolism Unit, Maria Vittoria Hospital, ASL TO2, 10144 Turin, Italy; ^2^Surgery Unit, Maria Vittoria Hospital, ASL TO2, 10144 Turin, Italy; ^3^Pathology Unit, Maria Vittoria Hospital, ASL TO2, 10144 Turin, Italy; ^4^3rd General and Esophageal Surgical Unit, University of Torino, 10126 Turin, Italy; ^5^Surgery Unit, San Giovanni Bosco Hospital, ASL TO2, 10154 Turin, Italy

## Abstract

Thyroidal pain is usually due to subacute thyroiditis (SAT). In more severe forms prednisone doses up to 40 mg daily for 2-3 weeks are recommended. Recurrences occur rarely and restoration of steroid treatment cures the disease. Rarely, patients with Hashimoto's thyroiditis (HT) have thyroidal pain (painful HT, PHT). Differently from SAT, occasional PHT patients showed no benefit from medical treatment so that thyroidectomy was necessary. We report three patients who did not show clinical response to prolonged high dose prednisone treatment: a 50-year-old man, a 35-year-old woman, and a 33-year-old woman. Thyroidectomy was necessary, respectively, after nine-month treatment with 50 mg daily, two-month treatment with 75 mg daily, and one-month treatment with 50 mg daily. The two women were typical cases of PHT. Conversely, in the first patient, thyroid histology showed features of granulomatous thyroiditis, typical of SAT, without fibrosis or lymphocytic infiltration, typical of HT/PHT, coupled to undetectable serum anti-thyroid antibodies. Our data (1) suggest that not only PHT but also SAT may show resistance to steroid treatment and (2) confirm a previous observation in a single PHT patient that increasing prednisone doses above conventional maximal dosages may not be useful in these patients.

## 1. Introduction

Pain originating from the thyroid gland is usually due to subacute (or de Quervain's) thyroiditis (SAT), a transient inflammatory thyroid disease of unknown origin, probably viral. Other well known causes of thyroid pain include hemorrhage in a thyroid cyst, bacterial infection of the thyroid, or an enlarged malignant tumor. However patients with the most common form of thyroiditis, Hashimoto's thyroiditis (HT), rarely have thyroid pain, leading to the clinical picture of painful Hashimoto's thyroiditis (PHT).

SAT is a self-limiting disease. In more severe forms of the condition, corticosteroids will generally cause a rapid relief of symptoms within 24–48 h. Prednisone may be initiated in dosages up to 40 mg daily for 2-3 weeks, with a gradual reduction in dosage thereafter. Recurrences do appear in a small percentage of patients, necessitating restoration of a higher dose once again [1, 2]. Conversely, very rarely, patients with PHT have only temporary or no benefit at all from medical treatment and/or show repeated relapses so that thyroidectomy is necessary to relieve pain [3–7].

In this paper we report three new patients with painful thyroiditis in which prolonged treatment with prednisone doses higher than the maximal recommended dose for SAT was completely ineffective in relieving pain and other clinical symptoms; in these patients total thyroidectomy was performed and led to complete clinical remission. These patients were observed in 1992, 2012, and 2013. At the time the first patient (from now on called Case 1) was observed, file data of outpatients were not completely available. Conversely, Cases 2 and 3 belong to a cohort of 73 patients with painful thyroiditis observed in the years 1996–2013. Clinical data of these patients are reported for comparison. Histological characteristics of the cases are also reported.

## 2. Cohort of Patients with Painful Thyroiditis Observed in the Years 1996–2013

Ninety-six patients presented to our observation with the clinical suspicion of painful thyroiditis in the years 1996–2013. We have considered these inclusion criteria: (1) pain clearly located in the thyroidal region, severe, spontaneous, and exacerbated by palpation, (2) increased erythrocyte sedimentation rate above 40 mm/h and/or elevated C reactive protein, (3) clinical and ultrasonographic exclusion of causes of thyroid pain other than thyroiditis, as those reported in the Introduction, and (4) thyroid ultrasonography consistent with thyroiditis, according to well established criteria [8, 9]. Some authors have individuated ultrasonographic and thyroid blood flow criteria useful to distinguish SAT from HT [10, 11]. These criteria were not applied since our main aim was to exclude patients without any form of thyroiditis. Moreover it is well known that ultrasonographic features of HT change during the natural history of the disease [8, 9] and a painful thyroiditis may happen in patients without thyroid antibodies, in others with positivity previously not known and of uncertain duration, and in others with well established autoimmune thyroiditis, associated or not with hypothyroidism. Therefore our inclusion criteria did not distinguish the possibilities of SAT and PHT. Including Cases 2 and 3, 73 patients matched all inclusion criteria, while other 23 were excluded since they did not. Follow-up of included patients ended in December 2014. Clinical details of included patients are reported in Table 1.

## 3. Case 1

**Figure 1:**
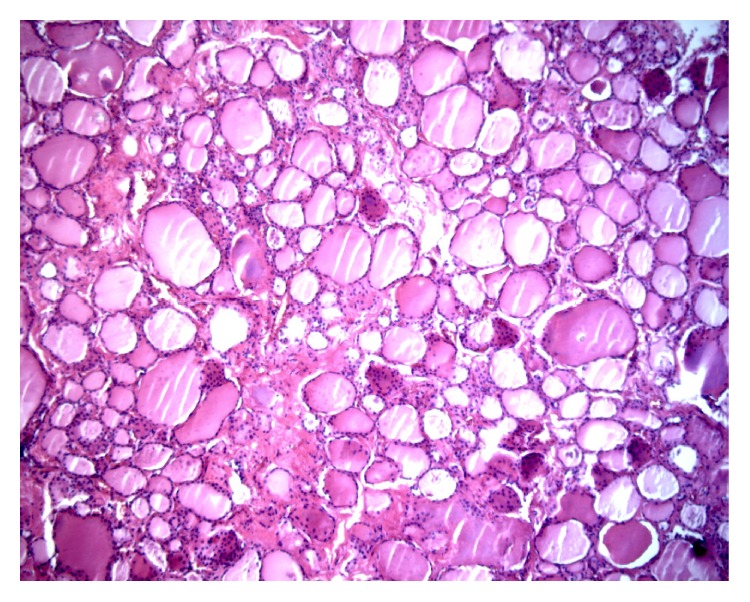
Case 1. Some granulomas with foreign body-type giant cells intermingled to medium-large size thyroid follicles with intraluminal colloid, lined by single layer of follicular cells.

A 50-year-old male originally from northern Italy presented to our observation in September 1992 with severe pain in the thyroidal region, spontaneous and exacerbated by palpation, low-grade fever, malaise, fatigue, and myalgias. A recent history of upper respiratory tract infection was present. Increased erythrocyte sedimentation rate (ESR) (94 mm/h) was present. Leucocytes' count was as follows: total 3890/mL, N 43.2%, L 51.3%, M 2.2%, E 3.0%, and B 0.3%, with absolute number of N being at the lower normal limit. Plasma protein electrophoresis showed a polyclonal increase of gamma globulin. fT3, fT4, and TSH levels were normal at the time of the diagnosis. Thyroid peroxidase antibodies (TPOAb), thyroglobulin antibodies (TgAb), and thyrotropin receptor antibodies (TRAb) were negative. Thyroid ultrasonography showed a slightly enlarged gland (A-P diameters, right lobe 18 mm, left lobe 19 mm); right lobe was slightly inhomogeneous, while left lobe showed a wide area of low echogenicity with shaded margins, occupying about 50% of the lobe, roughly corresponding to the most painful zone at the moment of ultrasonography; no thyroid nodule was observed. The patient was treated with prednisone 25 mg/day for 11 days, with no clinical bettering, so that the dose was increased to 40 mg/day, again with no clinical effectiveness. After another 10 days the dose was increased to 50 mg/day, with only partial clinical effectiveness; any attempt to reduce this drug dose throughout months always led to exacerbations of symptoms (particularly local pain but also systemic symptoms). On considering the unexpected resistance to steroid treatment, antibiotic therapy was empirically performed, even if at that time laboratory evaluation and ultrasonography were not in keeping with a bacterial infection of the thyroid. Leucocytes' count was as follows: total 8160/mL, N 63,6%, L 29,3%, M 2,8%, E 3,8%, and B 0,5%, with absolute number of the different types being normal. ESR was 26 mm/h; immunoglobulin and C3 and C4 assay were normal. No clinical effect was obtained. At that time thyroid ultrasonography showed a slightly enlarged gland with two shaded hypoechoic areas in the lower part of the right lobe and in the upper part of the left lobe. Computed tomography of the neck and chest was normal. ESR evaluated during the course of the disease was always above 25. fT3, fT4, and TSH were periodically evaluated during the course of the disease, remaining always normal; also TPOAb and TgAb levels remain undetectable, though they were reevaluated only during steroid treatment. A typical clinical picture of iatrogenic Cushing's syndrome, associated with overt secondary diabetes mellitus and hypokalemia, progressively developed and in July 1993 the patient was referred for thyroidectomy. The surgeon found difficulties due to adhesion of the gland to surrounding tissues, though no side effects of surgery happened. Total thyroidectomy led to complete resolution of pain. Steroid treatment was reduced and then stopped in about four weeks after surgery. The patient was on follow-up for at least 5 years without relapse of clinical symptoms. Besides, no clinical history suggesting immunological disorders as well as no major clinical problem was recorded, both before our observation and in the subsequent follow-up. Weight of the removed thyroid was 19 g. When Cases 2 and 3 were observed, histological slides of this patient were retrieved from archive and, after removal of cover slide, decoloured, restained with hematoxylin and eosin, and reevaluated. Histological examination showed the typical histological picture of granulomatous thyroiditis without any lymphocytic infiltration or fibrosis into the parenchyma. No clear evidence of capsular tissue fibrosis or thickening was evident in the available histological slides (Figure 1).

## 4. Case 2

**Figure 2:**
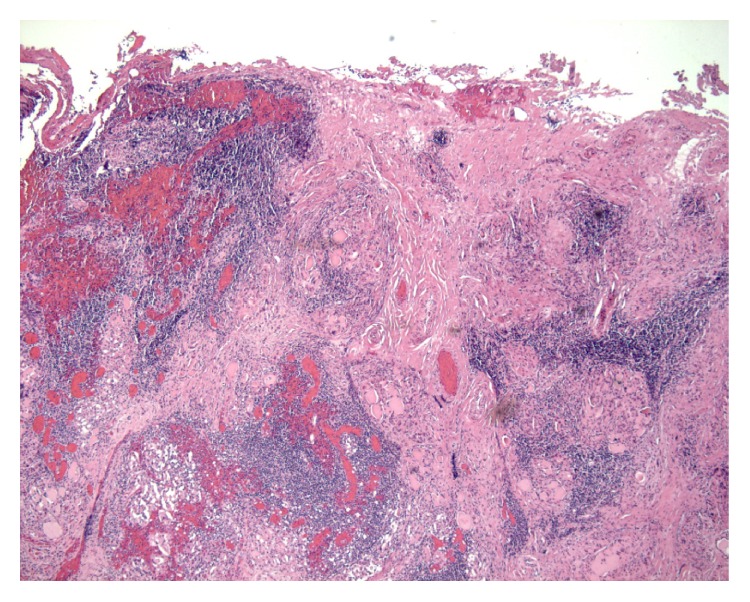
Case 2. Rare atrophic follicles with intraluminal colloid. Marked inflammation with extensive lymphocytic infiltration with some germinal centres and associated peripheral fibrous septa.

A 35-year-old woman originally from Morocco presented to our observation in May 2012 with severe thyroid pain, spontaneous and exacerbated by palpation, fever, malaise, and fatigue. A recent history of upper respiratory tract infection was not evident. She had primary autoimmune hypothyroidism known from about 5 years, well compensated with levothyroxine 1.2 mcg/kg. Increased ESR (88 mm/h) and slightly increased C reactive protein (CRP) (0.8 mg/dL, v.n. < 0.5) were present. Leucocytes' count was as follows: total 4890/mL, N 68.9%, L 26.7%, M 4.2%, E 0.2%, and B 0,0%, with absolute number of the different types being normal. Plasma protein electrophoresis was normal. Thyroid ultrasonography showed a gland slightly reduced in size (A-P diameters, right lobe 12 mm, left lobe 13 mm) with diffuse reduced echogenicity and hyperechoic fibrous septa; vascularization of the gland was not increased; no thyroid nodules were observed. Ultrasonographic examination of the thyroid was painful. Treatment with prednisone 37,5 mg/day was initiated with no clinical effectiveness, so that the drug dose was increased to 50 mg/day after 24 days and subsequently to 75 mg/day after another 29 days. ESR fell until 16 mm/h but clinical symptoms particularly spontaneous pain persisted. In this patient pain was periodically evaluated with visual analogic scale, remaining always above 7. Also in this case an antibiotic treatment was empirically performed. Just before this treatment leucocytes' count was as follows: total 10700/mL, N 69.0%, L 26.2%, M 4.8%, E 0.0%, B 0.1%, and CRP 0.17 mg/dL; thyroid ultrasonography was unchanged, computed tomography of neck and chest did not show other disorders. No clinical improvement was obtained after this treatment. In September 2012, after 60 days of prednisone 75 mg/day, and after hyperglycemia, hypokalemia, and progressive typical side effects of exogenous steroid became evident, total thyroidectomy was performed. Also in this case the surgeon found difficulties due to adhesion of the gland to surrounding tissues, though no side effects of surgery happened. Clinical remission of the disease was immediately obtained; steroid treatment was reduced and then stopped within three weeks. The patient is on follow-up and after 14 months we have not recorded relapse of clinical symptoms. Besides, no clinical history suggesting immunological disorders as well as no major clinical problem were recorded, both before our observation and in the subsequent follow-up. Weight of the removed thyroid was 11 g. Histological examination showed intense and extensive lymphocytic infiltration, marked thickening and fibrosis of the capsule and intrathyroidal septa, and no granuloma suggesting SAT (Figure 2).

## 5. Case 3

**Figure 3:**
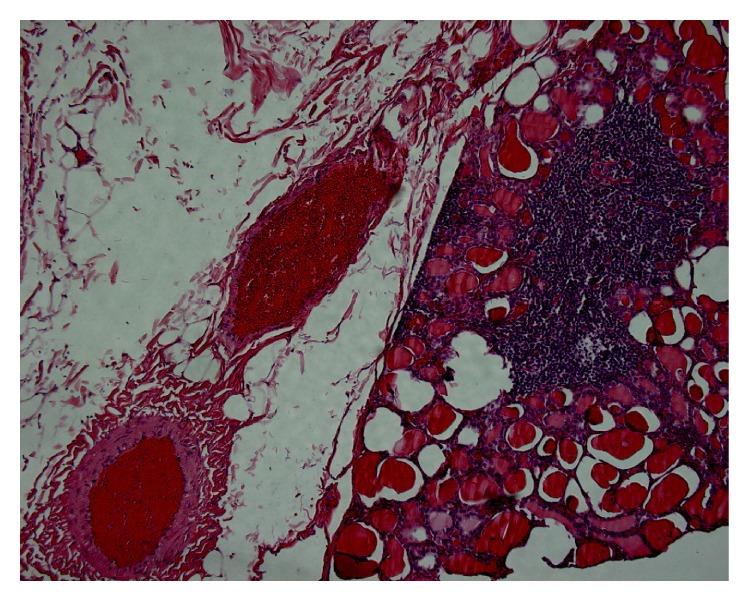
Case 3. Subcapsular nodular lymphocytic infiltration, medium-large size thyroid follicles with intraluminal colloid. On the left side perithyroidal adipose tissue with congested vessels.

A 24-year-old woman originally from northern Italy presented to our observation in June 2004 with thyroid pain, spontaneous and exacerbated by palpation. Increased ESR (61 mm/h) was present. She was euthyroid with increased TPOAb (256 U/L, normal values <10) and TgAb (86 U/L, normal values < 30) levels. Thyroid ultrasonography was consistent with thyroiditis with no thyroid nodules. Treatment with prednisone 12,5 mg/day was initiated and thereafter gradually withdrawn after 10 days. Relapses were observed in December 2004 (treated with prednisone 25 mg for 10 days with subsequent gradual withdrawal), August 2006 (prednisone 37,5 mg and then 40 mg for 93 days and thereafter gradual withdrawal through about two months), and May 2008 (prednisone 37,5 mg for 15 days and thereafter gradual withdrawal). TPOAb were found to be undetectable in the absence of steroid treatment in May 2008 (at the moment of the second relapse just before beginning of the steroid treatment). She had a successful pregnancy with delivery in May 2011. A new very painful relapse was observed in October 2013, accompanied by arthralgias, myalgias, and weakness; a recent history of upper respiratory tract infection was not evident. ESR was 32 mm/h and CRP 1.5 mg/dL. Leucocytes' count was as follows: total 8760/mL, N 64.9%, L 26.7%, M 5.4%, E 2.6%, and B 0,2%, with absolute number of the different types being normal. Plasma protein electrophoresis was normal. Control of thyroid ultrasonography was similar to the initial assessment, showing a normal size gland (A-P diameters, right lobe 15 mm, left lobe 16 mm); many hypoechoic pseudonodules were present, most of them with vascularization increased with respect to the surrounding thyroid tissue; again no thyroid nodules were observed. Ultrasonographic examination of the thyroid was painful. The patient was treated initially with prednisone 40 mg daily. No clinical response (documented by evaluation on visual analogic pain scale) was observed and drug dose was increased to 50 mg daily after 18 days. Thyroid antibodies were evaluated while the patient was taking this steroid dose: TPOAb were slightly elevated, (47 U/mL) while TgAb were normal (18 U/L) and TRAb were undetectable. Pain and systemic symptoms persisted without any improvement and after 31 days the patient underwent total thyroidectomy in December 2013. No main technical difficulties were encountered during surgery. Clinical remission of symptoms was immediately obtained and steroid treatment was withdrawn within two weeks. The patient is on follow-up and after six months we have not recorded relapse of clinical symptoms. Before thyroidectomy the patients remained euthyroid throughout the observation period and did not need thyroxin supplementation even in pregnancy, according to present guidelines. Besides, no clinical history suggesting immunological disorders as well as no major clinical problem was recorded, both before our observation and in the subsequent follow-up. Weight of the removed thyroid was 17 g. Histological examination showed lymphocytic infiltration, no granuloma suggesting SAT, and absence of significant fibrosis (Figure 3).

## 6. Discussion

SAT is a transient inflammatory thyroid disease of unknown origin, probably viral. It is clinically characterized by pain, usually severe, fever and other generalized systemic symptoms, increased erythrocyte sedimentation rate (ESR) or other markers of inflammation, and transient thyrotoxicosis due to gland follicle destruction, without true hyperthyroidism. Incomplete clinical forms are present. Treatment is based on salicylates and nonsteroidal anti-inflammatory drugs in patients with milder forms of the disorder. In more severe forms of the condition, corticosteroids will generally cause a rapid relief of symptoms within 24–48 h. Prednisone may be initiated in dosages up to 40 mg daily for 2-3 weeks, with a gradual reduction in dosage thereafter. Recurrences do appear in a small percentage of patients, necessitating restoration of a higher dose once again. This therapeutic approach is clearly accepted in medical literature [1, 2]. Follow-up and clinical outcome of patients with SAT are reported in some recent and extensive retrospective studies, covering almost 5,000 patients observed from 1970s on [12–17]; in these patients, the usually recommended dose of prednisone was always effective, even if prolonged treatment may be needed and relapses may occur (though rarely).

Patients with HT usually present with goiter, hypothyroidism, or both; in these patients some transient discomfort in the neck may be present due to thyroid enlargement, but thyroid pain is absent. Very few patients with HT have clinically relevant thyroid pain, often accompanied by systemic symptoms, such as those with SAT. However, differently from classical SAT, very rarely some of these patients have only temporary or no benefit at all from medical treatment and/or show repeated relapses so that thyroidectomy is necessary to relieve pain: up to now less than twenty such patients have been described [3–7]. The medical treatment was reported to be ineffective before the clinical decision for thyroidectomy; however it was not uniform. Most patients were treated with prednisone dosages ranging from 10 to 30 mg daily, only one patient with 40 mg/daily (i.e., the recommended dose for patients with clinically relevant painful SAT) [5] and another patient with higher doses, up to 70 mg/daily [4]. Treatment schedules of these patients were summarized in Table 2. Another patient was treated empirically with intrathyroidal steroid injections before surgery and was not considered in this paper [18], also cited in [19].

In this paper we report three cases of painful thyroiditis, observed over 21 years, who did not show any clinical response to prolonged treatment with prednisone doses higher than the maximal recommended dose for SAT (40 mg daily): about nine months with 50 mg daily in Case 1, two months with 75 mg daily in Case 2, and one month with 50 mg daily in Case 3. Total thyroidectomy was performed and led to complete clinical remission.

Cases 2 and 3 appear to be typical cases of PHT. Conversely Case 1 showed histological features of granulomatous thyroiditis without any evidence of fibrosis or lymphocytic infiltration, typical of HT/PHT. In this patient there were also undetectable serum levels of TPOAb and HTGAb. To our knowledge resistance to steroid treatment in an otherwise typical SAT has never been reported in the literature and suggests the possibility that patients not only with PHT but also with SAT may show resistance to steroid treatment.

Reportedly, the histological picture of SAT is characterized by granulomatous changes and giant cell formation, without extensive lymphocytic infiltration (so called granulomatous thyroiditis). Nowadays, after the introduction of steroid treatment, SAT has a benign course; therefore the statement of the correspondence of clinical SAT with the histological pattern of granulomatous thyroiditis is mainly based on the original observation [20] and on studies carried out in years in which the treatment of this disease was not based on steroids (see [21–23] as examples of studies covering a relatively wide number of patients). In relatively recent years Ishihara et al. [19] obtained a diagnosis of granulomatous thyroiditis with thyroid biopsy in 15 patients with clinical presentation consistent with SAT; there are to our knowledge only two relatively recent major extensive studies [24, 25] in which the diagnosis of subacute granulomatous thyroiditis was made* a posteriori, *based on the histological picture and independently of the clinical characteristics of the patients. These studies included 48 patients and it is clearly reported in the papers that none of them underwent thyroidectomy for relieving clinical symptoms, but for other reasons, mainly suspicion of malignancy or compressive nodular goiter.

On the contrary, all reported cases of PHT operated for untreatable thyroid pain [3–7] showed a thyroid histology similar to classical HT, characterized by extensive lymphocytic infiltration and fibrosis. None of them was reported to have granulomas and giant cells typical of SAT.

Conversely, few studies reported the possibility of granulomatous inflammation (always associated with lymphocytic infiltration) in bioptic thyroid specimen of patients with painful thyroiditis and positive anti-thyroid antibodies. These studies were essentially aimed at obtaining a definite diagnosis of PHT in patients with some degree of resistance to medical treatment. Shigemasa et al. [26] and Onoda et al. [7] reported histological changes of HT in 8/8 and 1/1 cases, respectively, without evidence of granulomatous changes. Kon and DeGroot [5] performed biopsy in 5 of their 7 patients with painful thyroiditis who subsequently underwent surgery for unremitting pain. In 2 of them “occasional giant cells” were reported, while granulomatous inflammation or giant cells were not reported in the final histological report of the surgical specimen. Ohye et al. [27] reported 2 patients: one of them showed both lymphocytic infiltration and granulomatous inflammation, while the other had only chronic lymphocytic thyroiditis. These observations (though only in 1/16 cases, with other 2 doubtful cases) may indicate that granulomatous changes may be present in PHT and are not specific for SAT. Alternatively, different mechanisms of painful thyroid inflammation may coexist in the same patient.

The positivity of thyroid antibodies is very prevalent in the general population [28]. Therefore SAT may occur in patients either without or with HT. The differential diagnosis SAT versus PHT is uncertain on clinical and laboratory basis [5]. Though not well stated in the studies, these appear to be the reasons why reported patients with PHT were initially treated with steroids and this is also the reason why we treated our patients subsequently found to be unresponsive to steroid treatment.

We speculate that patients with thyroid pain, ultrasonographic evidence of thyroiditis, positive anti-thyroid antibodies, and a good clinical response to steroids may have either mild PHT or SAT associated with HT. In our patients with clinical presentation substantially concordant with SAT the prevalence of cases with positive anti-thyroid antibodies was similar to that observed in the general population [28]. This could indicate that in most of them the clinical picture was unrelated to the presence of these antibodies and that their positivity is not sufficient for a diagnosis of PHT.

Similar to HT that is almost always painless, also SAT may be painless (and therefore may be not clinically relevant and undiagnosed): in the two previously cited major recent studies in which a histological diagnosis of subacute granulomatous thyroiditis was made* a posteriori *[24, 25], only about one-third of the patients (15/48) had clinical symptoms of SAT before surgery.

Thyroid antibodies assay in patients with thyroid pain has clinical interest considering the possible evolution of HT either in hypothyroidism or in hyperthyroidism. Conversely the differential diagnosis SAT versus PHT does not appear to have clinical relevance with respect to treatment of pain in the absolute majority of patients. This diagnosis may have some clinical interest in the very rare eventuality in which the response to maximal dose steroid treatment is poor and thyroidectomy has to be considered. In this context, as evidenced before, it is unclear whether thyroid biopsy has a clinical role. The role of ultrasonography is also undetermined. To our knowledge, only one paper was dedicated to this point: Onoda et al. [7] reported increased blood flow in hypoechoic lesions (though transiently) in 2/2 patients with steroid-resistant PHT confirmed at histology; this ultrasonographic pattern may be different from typical SAT [10, 11].

An interesting clinical feature of our three patients is that we have tried to overcome steroid resistance with prednisone doses higher than the maximal dose reported to be effective in SAT. Such doses were used only in one reported patient operated for PHT [4]. Our data reinforce this observation, suggesting that increasing prednisone doses above 40 mg daily in patients with painful thyroiditis (should this be SAT or PHT) could not have clinical utility. Obviously this statement needs to be confirmed, though the absent clinical response to steroids up to now appears to be extremely rare. In this context, evaluation of pain on a dedicated scale (as we have done in Cases 2 and 3) may also be proper to justify this unusual indication for thyroidectomy, also considering technical difficulties that may be encountered.

## Figures and Tables

**Table 1:** Seventy-three patients with painful thyroiditis observed in the years 1996–2013; clinical and laboratory details^*∗*^.

Clinical details	Subgroups	Data^*∗∗*^
Age; years, range, and (median)		14.4–75.2 (42.8)

Sex	Females	58
	Males	15

Systemic symptoms and/or fever	Yes	53
	No	20

Transient thyrotoxicosis	Yes	44
	No	29

TPOAb and/or TgAb levels	Above reference range	19
	Absent and/or within reference range	43
	Unknown	11

TRAb in patients with transient thyrotoxic	Positive	0
	Negative	35
	Unknown	9

Thyroid function in 43 cases with absent or normal TPOAb and TgAb	Transient thyrotoxicosis	28
	Euthyroidism during the observation period	15

Thyroid function in 19 patients with positive TPOAb and/or TgAb	Transient thyrotoxicosis	9
	Euthyroidism during the observation period	6
	Already on treatment with thyroxine for hypothyroidism	3
	euthyroid → Graves' disease^*∗∗∗*^	1

Thyroid nodules at ultrasonography	Yes	22
	No	49

Treatment schedule and outcome^*∗∗∗∗*^	Responders to salicylates or NSAIDs	11
	Responders to steroids^*∗∗∗∗∗*^	60
	Not responders to steroids (this paper, Cases 2 and 3)	2

^*∗*^Data are expressed in number of subjects when not otherwise specified in the column “clinical details.”

^*∗∗*^ TPOAb: thyroid peroxidase antibodies; TgAb: thyroglobulin antibodies; TRAb: thyrotropin receptor antibodies.

^*∗∗∗*^This patient was euthyroid during the painful phase; about three months after the resolution of painful thyroiditis the patient developed persistent thyrotoxicosis with positive TRAb, necessitating treatment with an antithyroid drug.

^*∗∗∗∗*^Before our observation, 18 patients were unsuccessfully treated with antibiotics; steroids were also sometimes used before our observation, but at doses lower than those subsequently found to be effective.

^*∗∗∗∗∗*^Patients were treated with prednisone 10–40 mg daily (58 cases) or other steroids in equivalent doses (2 cases). After 7–35 days (median 16) a gradual reduction in dosage could be initiated. Remission occurred in 57 patients. During the period of reduction of dosage or after discontinuation a single relapse was observed in 3 cases, all controlled with restoration of higher steroid dosage.

**Table 2:** Seventeen patients with PHT reported in the literature, who underwent thyroidectomy for thyroid pain resistant to medical treatment: treatment schedules before thyroidectomy.

Study	Number of cases	Treatment in each case (daily doses when specified) (see footnotes for abbreviations)
Zimmerman et al. 1986 [3]	2	NSAID + CS, NSAID + CS
Gourgiotis et al. 2002 [4]	2	P (70 mg), treatment not specified
Kon and DeGroot 2003 [5]	7	P (30 mg), P (40 mg), P (30 mg), P (30 mg), NSAID, NSAID, NSAID + P
Ohye et al. 2005 [6]	4	CS (15 mg), CS (10 mg), CS (15 mg), CS (20 mg)
Onoda et al. 2009 [7]	2	P (30 mg), PL (30 mg)

CS: corticosteroids, not specified.

ITCS: repeated intrathyroidal corticosteroid administration.

NSAID: nonsteroidal anti-inflammatory drugs and/or aspirin.

P: prednisone.

PL: prednisolone.
